# Switchmate! An Electrophysiological Attempt to Adjudicate Between Competing Accounts of Adjective-Noun Code-Switching

**DOI:** 10.3389/fpsyg.2020.549762

**Published:** 2020-11-17

**Authors:** Awel Vaughan-Evans, Maria Carmen Parafita Couto, Bastien Boutonnet, Noriko Hoshino, Peredur Webb-Davies, Margaret Deuchar, Guillaume Thierry

**Affiliations:** ^1^School of Psychology, Bangor University, Bangor, United Kingdom; ^2^Leiden University Centre for Linguistics, Leiden, Netherlands; ^3^Leiden Institute for Brain and Cognition, Leiden, Netherlands; ^4^Department of English, Tsuda University, Kodaira, Japan; ^5^School of Languages, Literatures, Linguistics and Media, Bangor University, Bangor, United Kingdom; ^6^Cambridge Language Sciences, University of Cambridge, Cambridge, United Kingdom; ^7^Centre for Research on Bilingualism, Bangor University, Bangor, United Kingdom

**Keywords:** code-switching, Minimalist Programme, matrix language framework, word order, bilingualism, Welsh, English, event-related brain potentials

## Abstract

Here, we used event-related potentials to test the predictions of two prominent accounts of code-switching in bilinguals: The Matrix Language Framework (MLF; Myers-Scotton, 1993) and an application of the Minimalist Programme (MP; Cantone and MacSwan, 2009). We focused on the relative order of the noun with respect to the adjective in mixed Welsh–English nominal constructions given the clear contrast between pre- and post-nominal adjective position between Welsh and English. MP would predict that the language of the adjective should determine felicitous word order (i.e., English adjectives should appear pre-nominally and Welsh adjectives post-nominally). In contrast, MLF contends that it is the language of the finite verb inflexion rather than that of a particular word that governs felicitous word order. To assess the predictions of the two models, we constructed sentences featuring a code-switch between the adjective and the noun, that complied with either English or Welsh word-order. Highly proficient Welsh–English bilinguals made semantic acceptability judgements upon reading the last word of sentences which could violate MP assumptions, MLF assumptions, both assumptions, or neither. Behaviourally, MP violations had no significant effect, whereas MLF violations induced an average drop of 11% in acceptability judgements. Neurophysiologically, MP violations elicited a significant Left Anterior Negativity (LAN) modulation, whereas MLF violations modulated both P600 and LAN mean amplitudes. In addition, there was a significant interaction between MP and MLF status in the P600 range: When MP was violated, MLF status did not matter, and when MP criteria were met, MLF violations resulted in a P600 modulation. This interaction possibly reflects a general preference for noun over adjective insertions, and may provide support for MLF over MP at a global sentence processing level. Model predictions also manifested differently in each of the matrix languages (MLs): When the ML was Welsh, MP and MLF violations elicited greater P600 mean amplitudes than MP and MLF adherences, however, this pattern was not observed when the ML was English. We discuss methodological considerations relating to the neuroscientific study of code-switching, and the extent to which our results shed light on adjective-noun code-switching beyond findings from production and experimental-behavioural studies.

## Introduction

It is common for bilinguals to mix their languages in the same sentence or conversation. This phenomenon is known as code-switching (Deuchar, 2012). In this study, we focus on switching where the structures of the two languages differ (conflict sites). We selectively target adjective-noun switches in a language pair, Welsh-English, where adjectives are pre-nominal in one of the languages (English ¨red wine¨) and post-nominal in the other language (Welsh ¨gwin coch¨ -*wine red*). Thus, Welsh-English code-switching between the noun and the adjective could generate four potential noun-adjective combinations: ‘red gwin,’ ‘gwin red,’ ‘coch wine,’ and ‘wine coch’. In general, Welsh–English code-switching data show clear regularities, with Welsh grammar determining word order in bilingual clauses with very few exceptions (see Deuchar et al., 2018). However, due to the generally low occurrence of attributive adjectives in production data, determining the grammatical constraints that may predict code-switching patterns has been the focus of attention of many studies, not only on Welsh–English code-switching (Parafita Couto et al., 2015, 2017) but also on adjective-noun code-switching in other language pairs where the switch point also constitutes a conflict site (e.g., Spanish–English, Papiamento–Dutch, or French–Dutch). Most of these studies examined patterns of adjective-noun switching in different bilingual populations and using different methodologies, to evaluate the predictions of two theoretical accounts: the Matrix Language Framework (MLF, Myers-Scotton, 1993) and the Minimalist Programme approach (MP, Cantone and MacSwan, 2009). An overview of these studies is provided in section “Previous studies evaluating the predictions of the MLF vs. MP,” but we first provide a brief review of the theoretical accounts that we are testing.

### The Matrix Language Framework (MLF)

According to proponents of the MLF (Myers-Scotton, 1993, 2002), the grammar of one of the two languages in the bilingual clause takes priority. A distinction is drawn between the ‘matrix language’ (ML), which provides the morphosyntactic frame for the clause, and the ‘embedded language’ (EL), which provides embedded elements, mainly content words. The MLF predicts that (i) finite verb morphology and (ii) word order within a clause will be sourced from the same language (the ML). If the finite verb morphology is from one language, then the prediction is for the relative word order within the adjective-noun phrase to also be from that language. This means that in a sentence with Welsh as ML, the adjective will be postnominal irrespective of the language of the adjective (Welsh or English), as in (1a,b; note that the code-switches are highlighted in bold). Conversely, in a sentence with English as ML (i.e., when the finite verb of the clause is in English), the adjective will be prenominal independently of whether the adjective comes from Welsh or English, as in (2a,b; note that the code-switches are highlighted in bold).


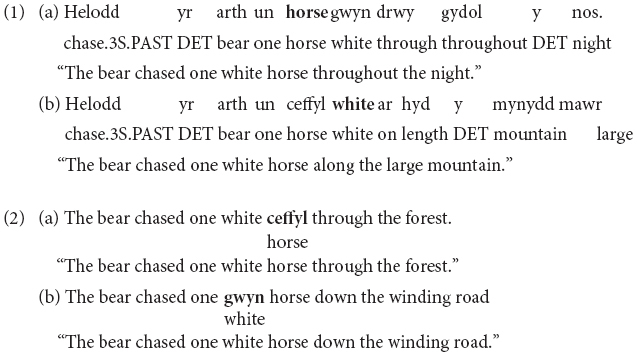


### Minimalist Programme (MP)

In contrast to the proponents of the MLF approach, those who support the MP seek to explain code-switching using exclusively the grammatical features of the participating languages (MacSwan, 1999). MacSwan (1999) criticised the MLF, arguing that this framework explicitly refers to the separate languages involved in it. He argues that code-switching data should be explained in the same way we explain monolingual grammars. Regarding specific predictions for adjective-noun order, Cantone and MacSwan (2009) follow Cinque’s (1994, 1999, 2005) proposal that adjectives universally precede nouns in their exploration of Italian–German spontaneous data. Under this view, differences in word order between languages (like Italian and German, or English and Welsh) follow from overt movement of the noun (Welsh or Italian) to a position to the left of the adjective. This noun movement results in postnominal adjective order in those languages. They reach the descriptive generalisation that “while the data remain slightly ambiguous, a relatively clear pattern has emerged in both the survey data and the naturalistic data confirming the general view of previous researchers, namely, that the word order requirements of the language of the adjective determine word order in code-switching in DP-internal contexts” (Cantone and MacSwan, 2009, pp. 266–267). This means that whenever the adjective is English it will be prenominal (3a,b; note that the code-switches are highlighted in bold), and whenever it is Welsh it will be postnominal (4a,b; note that the code-switches are highlighted in bold), independently of the ML of the clause.


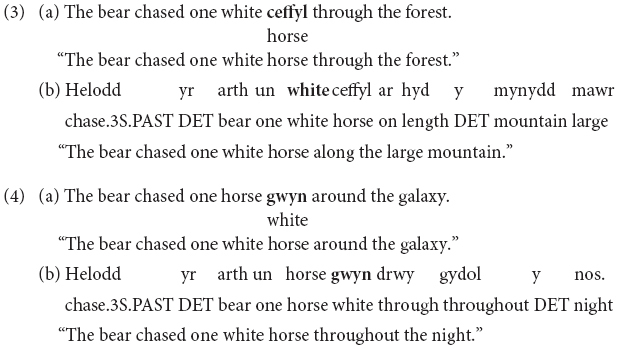


### Previous Studies Evaluating the Predictions of the MLF vs. MP

In this section, we review studies that tested the predictions of the MLF against the MP predictions of Cantone and MacSwan (2009) for adjective-noun switching. These studies were conducted on different language pairs and also used different methodologies: production data (naturalistic or elicited), acceptability judgment tasks (AJTs), electrophysiological measures, or a combination thereof. In what follows, we will first discuss the literature using production data, then we will focus on studies that used AJTs and finally we will provide details on the two previous neurocognitive studies on adjective-noun code-switching.

#### Production Data

Production data and corpus analyses can provide a wealth of information about the naturalistic occurrences of code-switches and allow for the predictions of both MLF and MP to be assessed in an ecologically valid way. Parafita Couto et al. (2015) used a multitask approach comprising two sources of production data (naturalistic corpus data and data from an elicitation task), and an auditory judgement task to investigate the contrasting predictions of both models in Welsh–English bilingual speech. In their production data, Welsh was the ML for all sentences, and the most common code-switched combinations included an English noun followed by a Welsh adjective. Whilst these data provide valuable insight into a preference for noun-insertions over adjective insertions, they cannot be used to contrast the predictions of each model, as such insertions correspond with both the predictions of the MLF and MP. The authors therefore focused on mixed nominal constructions with non-ML adjectives, as both models make contrasting predictions in this case (the MLF predicts adjectives to follow the order of the ML, while the MP predicts adjectives to follow the word order of their language origin). A total of 43 mixed nominal constructions were identified in the naturalistic conversational corpus, with seven adhering to the predictions of MP, and 36 adhering to the predictions of MLF. A similar pattern was observed in the elicited data, thus providing tentative support for MLF over MP. In contrast, participants rejected all items during the judgement task, which may reflect a stigma associated with code-switched utterances in this population (but see section “Acceptability Judgements” for further discussion on judgement/acceptability tasks). Overall, these data highlight a preference for switched words to be nouns rather than adjectives, resulting in the predictions of both theoretical accounts being adhered to in most occurrences of adjective-noun switches in this study.

Support for both theoretical approaches was also provided by Parafita Couto and Gullberg (2019), who examined code-switched determiner-noun-adjective complexes in three language pairs (Welsh–English, Spanish–English, and Papiamento–Dutch). They extracted all mixed nominal constructions including an adjective [determiner–adjective–noun (DetAN/NA)]. The most common pattern is for determiners in Welsh, Spanish, and Papiamento to be followed by adjective-noun clusters in English and Dutch, with adjectives in the typical prenominal position of these languages. These findings adhere to MP predictions, but arguably also the MLF predictions, as the MLF allows for “EL islands” where the grammar of the EL prevails (Myers-Scotton, 1993). Such adjective-noun combinations from the EL would be considered ‘EL islands.’ Overall, these findings suggest that switches predominantly occur between Determiners and Adjective Noun clusters or “EL islands” [e.g., Welsh–English “y Belgian loaf” (the._Welsh_ Belgian loaf)], not between Adjectives and Nouns, and provide support for both theoretical accounts. However, in the nine examples of switches between adjectives and nouns, the adjective position always matched the ML, thus providing tentative support for MLF over MP.

Balam and Parafita Couto (2019) extended this line of research to a different Spanish–English bilingual community: Northern Belize. They extracted 1680 nominal constructions (477 monolingual Spanish and 1203 Spanish–English) from sociolinguistic interviews with 62 Spanish–English bilinguals from Northern Belize. Their analysis showed that bilinguals avoid Spanish attributive adjectives and overt gender marking in mixed nominal constructions, but not in monolingual Spanish ones. This pattern in the data is explained by Otheguy and Lapidus’ (2003) adaptive simplification hypothesis, which posits that bilinguals avoid switching in grammatical contexts where gender marking is required. In terms of adjective placement, again both the MLF and Cantone and MacSwan’s MP predictions were able to account for mixed noun-adjective constructions, with only a few exceptions that could only be predicted by the MLF. Similar to what was reported by Parafita Couto and Gullberg (2019), Balam and Parafita Couto’s (2019) results also revealed that most adjectival constructions in code-switching contained embedded language islands (88.8%). Additionally, mixed nominal constructions containing a gender-marked Spanish attributive adjective were not common in their data. Consonant with the findings of the two previous studies, the Northern Belize data point in the direction of the relative superiority of the MLF.

The findings of these studies provide insights into code-switching patterns in naturalistic production data. Specifically, they highlight the infrequent use of noun-adjective switches within nominal constructions (see Pfaff, 1979; Poplack, 1980; Otheguy and Lapidus, 2003 for similar findings), and a general preference for noun insertions over adjective insertions. However, these patterns do not allow for a direct comparison of the contrasting model predictions, as they generally adhere to the predictions made by both models. In addition, the data analysed in these studies mainly consisted of sentences in one ML (e.g., Welsh for Welsh–English bilinguals), and so it is unclear whether similar patterns would emerge in the other ML (e.g., English). Additional research is needed to directly contrast the predictions of each model.

#### Acceptability Judgements

Despite the descriptive richness and ecological validity of naturalistic production data, some researchers argue that corpus data also has inherent limitations as counterexamples may exist that are not attested in the corpus (see Gullberg et al., 2009, for an overview). Many linguistic studies use acceptability judgments tasks (AJTs; for a review, see Schütze, 2016), where participants indicate whether a sentence is grammatically correct or acceptable, or specify the degree of acceptability on a given scale.

Vanden Wyngaerd (2017) examined the predictions made by an MP approach (Cantone and MacSwan, 2009) and the MLF regarding both word order as well as adjectival agreement patterns in French–Dutch mixed nominal constructions. In Dutch, adjectives occur pre-nominally, whilst adjectives are predominantly post-nominal in French, thus allowing for a direct contrast of both models. Vanden Wyngaerd used a 3-point acceptability task in which she orally presented 120 code-switched sentences to 15 bilingual participants. Overall, her findings indicate that the MP is better at predicting grammaticality than the MLF, as sentences that adhered to the predictions of MP were rated as more acceptable than sentences that violated the predictions of MP. Interestingly, there was no statistically significant difference in the mean rating of sentences with a Dutch ML and a French adjective, and sentences with a French ML and a Dutch adjective. This finding directly contrasts with previous findings by Treffers-Daller (1993), who noted, based on naturalistic data, that it is more common for Dutch adjectives to be inserted in a French sentence than the other way around. The author acknowledges that methodological differences in corpus and grammaticality judgement tasks may lead to divergent results and concludes that her findings in favour of the MP should be seen as provisory.

Stadthagen-González et al. (2017) also assessed the predictions of MP and MLF in relation to adjective-noun word order in Spanish–English code-switched sentences (English has prenominal adjectives while Spanish prefers postnominal adjectives). They constructed sentences containing code-switched adjective-noun phrases that adhered to the predictions of MLF, MP, both, or neither, and assessed the acceptability of the sentences in two separate experiments: one using a 5-point Likert scale, and one using a 2-Alternative Forced Choice (2AFC) task. The results from both tasks revealed an additive effect, as both the language of the verb inflexion and the language of the adjective determine word order [see Voss (2018) for similar findings in a sample of Papiamento–Dutch bilinguals]. Thus, they argue that neither the MLF nor the MP can completely explain the acceptability of adjective–noun switches and propose that progress in our understanding of grammaticality in code-switching will be accomplished by incorporating observations from the two frameworks rather than examining them separately.

These findings paint a complex picture, with neither model fully accounting for the reported results. They contrast with data from naturalistic corpus studies, and it must be noted that acceptability judgement tasks may not be suitable for code-switching research, particularly in communities where code-switching is stigmatised (cf. Stadthagen-González et al., 2018). It is possible, for example, that negative attitudes toward code-switching may lead participants to reject grammatical constructions that their linguistic systems would in fact permit (cf. Parafita Couto et al., 2015 for the specific case of Welsh–English).

#### Electrophysiological Measures

As Phillips et al. (2019) put it: “We often encounter the hope that experiments will give us more precise data that will allow us to settle difficult theoretical questions” (p. 1). It is with this hope that the studies reviewed in this section were conducted.

Given that code-switching is often stigmatised, behavioural investigations into the acceptability of code-switched utterances may be susceptible to stereotypical judgements. Alternative neuroscientific methods may overcome this constraint by measuring implicit responses that occur prior to conscious judgments (Parafita Couto et al., 2015). To date, few studies have used neuroscientific methods to investigate code-switching, and the majority of these studies have focused specifically on the costs associated with language switching (see Van Hell et al., 2015, 2018, for relevant reviews). These studies primarily focus on comparing lexical insertions with non-switched semantically congruent or incongruent completions (e.g., Moreno et al., 2002; Proverbio et al., 2004; Ruigendijk et al., 2016, but see Litcofsky and Van Hell, 2017 for an investigation of multi-word switches), and have provided a great deal of insight into the neurological correlates associated with code-switches. However, these studies did not explicitly test the grammaticality of the code-switches, which is the purpose of the current study. To our knowledge, only two ERP studies have explicitly tested the grammaticality of code-switches by directly comparing the predictions of two theoretical linguistic models (MLF and MP). Parafita Couto et al. (2017) conducted an electrophysiological study on Welsh-English code-switching, focusing specifically on adjective-noun switching. Their study contained four experimental sentence types (Table 1), and two critical contrasts were conducted.

**TABLE 1:** Example experimental sentences from Parafita Couto et al. (2017).

**Sentence**	**MLF**	**MP**
The bear chased one **gwyn** horse	+	–
Helodd yr arth un horse **gwyn**	+	+
The bear chased one ceffyl **white**	–	–
Helodd yr arth un **white** ceffyl	–	+

*The adjectives at which ERP responses were measured and analysed are highlighted in bold.*

The first contrast compared sentences where the models made orthogonal predictions (MLF+MP− vs. MLF−MP+) whilst the second compared sentences where both models were adhered to (MLF+MP+) with sentences that violated the predictions of both models (MLF−MP−). All contrasts focused on a negative-going ERP waveform corresponding to a left anterior negativity (LAN), an ERP component sensitive to phrase structure or morphosyntactic violations (e.g., Friederici et al., 1993; Hahne and Friederici, 1999; Friederici, 2002; Hagoort, 2003). The first contrast revealed that MLF−MP+ elicited more negative ERP amplitudes than MLF+MP− sentences, suggesting that MLF−MP+ sentences were more difficult to process than MLF+MP− sentences. This first contrast therefore provided tentative support for MLF over MP, however, the orthogonal predictions meant that no definite conclusions could be drawn. However, the second comparison did not yield any significant differences. It is possible that this null result was caused, in part, by sentence ‘wrap-up’ effects (Hagoort et al., 2003), given that the critical stimuli appeared in sentence-final position, even though recent findings cast doubt on this interpretation (e.g., Stowe et al., 2018). Nevertheless, the authors acknowledged this limitation and suggested that the inclusion of an adverbial or prepositional phrase at the end of the experimental sentences may help resolve the ambiguity of these results. Note, however, that the null effect in the second comparison may also have occurred due to carry-over effects from the preceding code-switch.

Building on the study by Parafita Couto et al. (2017), Pablos et al. (2018) tested the predictions of these models regarding noun-adjective order in Papiamento–Dutch code-switched utterances. Pablos et al. (2018) also tested the possibility that either word order may be possible in modification sites (Di Sciullo, 2014). They evaluated the predictions of the theoretical approaches using the same design as Parafita Couto et al. (2017). In contrast to Parafita Couto et al. (2017), they found no LAN modulation as a result of their experimental manipulations, and as such were unable to support the predictions of neither MLF nor MP. For monolingual non-switched control sentences, there was no significant difference between amplitudes elicited by Papiamento and Dutch adjectives. For code-switched sentences, the authors checked for effects at the adjective position in sentences on which the models made opposite predictions, but they found no evidence to indicate any differences between the ERPs elicited by the adjectives in these sentences. They also found no effect at the adjective position in sentences on which the models made similar predictions. Since there was no difference in responses, these results can either be interpreted as favouring Di Sciullo’s prediction that either order may be possible, or, as an indication of a rejection of all code-switched patterns.

ERP and corpus studies investigating the contrasting predictions of MLF and MP have thus far failed to provide conclusive evidence in support of either model. It is possible that these conflicting findings result from a fundamental difference in the processing mechanisms involved in production and comprehension. However, recent connectionist models in the psycholinguistics literature (e.g., MacDonald, 2013; Pickering and Garrod, 2013; Dell and Chang, 2014) dispel this suggestion, and convincingly demonstrate a cyclical link between comprehension and production. A close examination of the stimuli used in Parafita Couto et al. (2017) and Pablos et al. (2018) also support this link: No difference was observed between sentences that adhered to the predictions of both models and sentences that violated the predictions of both models in either of the studies. In each case, these sentences included noun insertions, which are arguably preferred over adjective insertions (Parafita Couto et al., 2015). In addition, the orthogonal predictions of the models were spread across two MLs for both studies, despite the production literature demonstrating that code-switching patterns in such bilingual populations typically occur in only one language (e.g., Welsh ML sentences for Welsh-English bilinguals; Parafita Couto et al., 2015, and Papiamento ML sentences for Papiamento-Dutch bilinguals; Parafita Couto and Gullberg, 2019). As such, the contrasting results of the two studies may reflect methodological differences, rather than fundamental differences in production and comprehension processes.

### Predictions for the Present Study

In the current study, we utilised electrophysiological and behavioural measures to further investigate the two competing theoretical models. Following on from previous studies, we chose to focus on adjective-noun constructions in Welsh-English bilinguals. Here, however, we included additional sentence conditions to capture a range of possible code-switches, thus allowing for a more in-depth analysis of the model predictions. We also adapted the stimuli of our previous study (Parafita Couto et al., 2017) to avoid potential ‘wrap-up’ effects, and incorporated a semantic acceptability task. This resulted in eight sentence types, each containing a code-switch within an adjective-noun construction: Four sentences were categorised as having English as the ML, and four were categorised as having Welsh as the ML (Table 2).

**TABLE 2:** Experimental design and stimulus examples.

**Sentence**	**MLF**	**MP**
The girl bought one bird **bach** from the pet store.	**–**	**+**
The girl bought one small **aderyn** *with her feet.*	**+**	**+**
The girl bought one **aderyn** small without telling her parents.	**–**	**–**
The girl bought one **bach** bird during a shopping spree.	**+**	**–**
Prynodd y ferch un **bird** bach gyda ei phres poced.	**+**	**+**
Prynodd y ferch un **small** aderyn ar ôl ysgol.	**–**	**+**
Prynodd y ferch un aderyn **small** fel anhreg i’w chwaer.	**+**	**–**
Prynodd y ferch un bach **bird** yn ystod gwyliau’r haf.	**–**	**–**

*The point at which a code-switch occurs is highlighted in bold. Predictions of violations made according to the two main theoretical frameworks based on the position of the *adjective*: + means adherence to the prediction and – means violation of the prediction. An example of a semantically aberrant sentence ending is highlighted in italics, though note that these incongruent completions were dispersed equally between conditions. Italics and bold font are for the reader’s information and were not used in the experiment.*

The predictions specified by each theoretical model encompass the adjective-noun phrase in its entirety. In our initial analysis, we measured event-related potentials (ERPs) elicited by the final word within the adjective-noun construction, and all our electrophysiological predictions related to the final word within that construction^1^. Given that half of our experimental sentences included a noun-adjective construction, and half included an adjective-noun construction, we analysed these sentences separately, and focused on two distinct ERP components to accurately evaluate the predictions of the two models.

In our first (planned) analysis, we investigated ERP responses elicited by critical nouns on the one hand and critical adjectives on the other, in the time window of the LAN: an ERP index considered to reflect early grammatical processing (Friederici et al., 1993), and in the time window of the P600: an ERP component typically involved in global grammatical processing and sentence re-evaluation (Osterhout and Holcomb, 1992; Steinhauer et al., 2009, 2010). This decision was made as the predictions of both models refer to the placement of the *adjective* within the construction, and as such ERP responses elicited by nouns would differ to ERP responses elicited by adjectives. Note that, whilst other studies have reported that morphosyntactic violations modulate N400 mean amplitude (e.g., Guajardo and Wicha, 2014; Lau et al., 2016), we focused on the LAN to ensure consistency with the two other ERP studies (Parafita Couto et al., 2017; Pablos et al., 2018) that have investigated the grammaticality of adjective-noun code-switching. If participants are sensitive to the predictions of MLF, constructions that violated its predictions should elicit more negative LAN mean amplitudes, and more positive P600 mean amplitudes, than constructions that adhered to its predictions. If participants are sensitive to the predictions of MP, a similar pattern should emerge. If LAN mean amplitudes are not modulated in line with the predictions of the models, then this would suggest that these predictions are not processed at a local, early grammatical processing stage. If P600 mean amplitudes are not modulated by the predictions of the models, then this would suggest that these predictions are not processed at a global, sentence-level processing stage.

In a second (extended) analysis, we compared ERPs elicited by the adjective within an adjective-noun construction, regardless of whether it occurred before or after the noun, to determine whether the model predictions were modulated by the ML of the sentence. Our predictions in this analysis are identical to the predictions outlined above.

A selection of the experimental sentences also included a semantically incongruent completion *after* the presentation of the adjective-noun construction. Participants were required to explicitly state whether the sentences ‘made sense’ upon reading the sentence-final word. This manipulation was included to ensure participant engagement, but also allowed for an indirect measure of the model predictions at a surface level. Our predictions regarding overt behavioural responses relate specifically to sentences that contain a semantically congruent completion. If participants are sensitive to the predictions of the models at a global, sentence processing level, sentences that adhere to the model predictions should be categorised as semantically acceptable *more* than sentences that violated the predictions of the models.

## Materials and Methods

### Participants

Twenty-six Welsh–English bilinguals participated in this study. Of this sample, one participant was removed due to low Welsh proficiency, three participants were removed as their EEG data contained too few epochs per condition, and a further four participants were excluded due to alpha contamination in the EEG data. Thus, 18 highly proficient participants (4 male; *M*_*age*_ = 22.11 years; *SD* = 4.30 years) were included in the final analysis, all of whom self-reported that they had learnt English from an early age (*M* = 2.82 years; *SD* = 2.88). Eight participants identified as simultaneous bilinguals, whilst ten participants identified Welsh as their native language, with English being acquired in an educational setting. Participants rated their reading and writing proficiency, their conversational fluency, and their speech comprehension in both languages, and their overall proficiency score did not differ significantly between Welsh (*M* = 9, *SD* = 0.97) and English (*M* = 9.13, *SD* = 1.08). All participants possessed normal or corrected to normal vision. Ethical approval was obtained from Bangor University Psychology Ethics Committee, and all participants provided written consent.

### Materials and Design

The stimuli comprised 32 sentence sets, with 8 sentences in each set. To create the experimental sentences, we first selected 16 subject nouns, 16 verbs, 16 object nouns, and 16 adjectives. These words were non-cognates, the object nouns included in the adjective-noun constructions were masculine (so as to avoid interference from the Welsh morphosyntactic rules of soft mutation; Ball and Müller, 1992), and each word appeared in two experimental sentences. Within a sentence set, four of the sentences had Welsh as a morphosyntactic frame, and four had English as a morphosyntactic frame. Furthermore, the order of the adjective-noun construction was altered in each sentence, and each sentence contained a code-switch (see Table 2). These manipulations ensured that each experimental sentence within a set either adhered to, or violated, the predictions of the competing models. Each sentence set also included a semantically ‘incongruent’ sentence completion, upon which the behavioural task was based. Crucially, this semantic manipulation occurred *after* the code-switch within each sentence and was rotated across all experimental conditions. This distractor task was used to draw the participants’ attention away from the experimental manipulation, whilst also allowing for an indirect measure of model predictions. For our planned analyses, the crucial experimental conditions included Welsh ML sentences and English ML sentences. We thus did not include ML as an experimental factor. The experiment therefore comprised a 2 (MLF+ vs. MLF−) × 2 (MP+ vs. MP−) repeated measures design, in which each participant viewed all sentence versions. In our extended analysis, however, we included ML as an experimental factor, resulting in a 2 (Matrix Language: English vs. Welsh) × 2 (MLF+ vs. MLF−) × 2 (MP+ vs. MP−) repeated measures design, in which each participant viewed all sentence versions.

### Procedure

Participants viewed all 256 sentences, presented in 18 point font on a black background. Sentences were segmented into nine sections before presentation. The first eight sections contained a single word, and were presented for 500 ms, with 200 ms ISI. The ninth section contained the remainder of the sentence, and was presented for 2000 ms, or until the participant made a response. The critical adjective-noun constructions appeared in segment five and six in all cases (e.g., The | girl | bought | one | bach | bird **|** during | a | shopping spree.)

The experiment comprised of 8 blocks, and presentation order was pseudorandomized, such that two sentences from a single sentence set were never presented in the same block. In addition, each block included sentences from every experimental condition. This decision also allowed us to control for potential repetition effects, as each condition would be equally impacted. At the end of each sentence, participants stated whether the sentence made sense, by means of a button box. This task was used to ensure participant engagement with the stimuli and focused on semantic rather than grammatical violations. Following the experiment, we obtained demographic information (age of acquisition, frequency of use, and native language) using a language history questionnaire. Participants also rated their reading and writing proficiency, their conversational fluency, and their comprehension ability for each language, and responses were averaged to generate an overall Welsh and English proficiency score for each participant.

### ERP Recording

Electrophysiological data were recorded from 64 Ag/AgCl electrodes according to the extended 10–20 convention and were referenced to Cz at a rate of 1 kHz. Impedances were kept < 5 k Ω and the electroencephalogram (EEG) activity was filtered online with a band-pass philtre between 0.05 and 200 Hz, and offline with a low-pass zero-phase shift digital philtre which was set at 25 Hz. The data were then pre-processed using MATLAB (R2014a, The Mathworks, Inc.), and the EEGLAB (Delorme and Makeig, 2004) and ERPLAB (Lopez-Calderon and Luck, 2014) toolboxes. The continuous EEG data was visually inspected, and excessive muscular artefacts were manually removed. Epochs ranging from −100 to 1000 ms from the onset of the target word were extracted from the EEG recordings, and an independent component analysis (ICA; e.g., Makeig and Onton, 2011) was performed to identify and extract remaining muscular and ocular artefacts. A maximum of five independent components were removed per participant. Epochs with activity exceeding ± 200 μV at any electrode site were automatically discarded. There was a minimum of 24 epochs per condition for every participant. Baseline correction was performed in reference to 100 ms of pre-stimulus activity, and individual averages were digitally re-referenced to the global average reference.

### Behavioural Data Analysis

Data were analysed using the lme4 package, version 1.1-12 (Bates et al., 2015) in R version 3.2.3 (R Core Team, 2015). Only sentences containing a semantically congruent completion (as determined by the experimenters prior to testing) were included in the analysis. For example, behavioural responses to the sentence ‘*The girl bought one small aderyn with her feet’* were not included in this analysis, as this was categorised as a semantically incongruent sentence prior to testing. Note that the semantically incongruent completions were evenly distributed across all conditions, and so all eight critical conditions were included in the analysis. These sentence types should all be perceived as semantically congruent to participants, and thus any differences that may arise could be attributed to our experimental manipulation. After excluding responses to semantically incongruent sentences, button responses were triaged into congruent (*yes, this sentence makes sense*) and incongruent (*no, this sentence doesn’t make sense*) responses from the participant’s point of view, hereafter subjective responses. In other words, all responses included in the analysis were collected in response to sentences that were semantically congruent but judged as congruent or incongruent by the participant (not predictions from MLF and MP).

Responses were analysed by means of a binomial logistic regression, and reaction time data were examined with linear mixed effects analyses. An interaction term for the two repeated measures factors (MLF^∗^MP) was included for both analyses, and the baseline (intercept) of each analysis comprised of the ‘MLF−’ and ‘MP−’ conditions. For the reaction time data, the ‘sentence’ variable was modelled as a function of intercept performance, whilst the ‘participant’ variable included the intercept, plus the maximal slope of MLF^∗^MP (Barr et al., 2013).

Treatment contrasts were used to interpret the model output, and the specifications of each model allowed for two fixed effects as well as one interaction term. Fixed Effect 1 compared ‘MP−’ trials in ‘MLF−’ and ‘MLF+’ conditions. Fixed Effect 2 compared ‘MP−’ trials with ‘MP+’ trials in ‘MLF−’ conditions. Finally, the Interaction assessed the extent to which differences in ‘MP−’ vs. ‘MP+’ trials were specifically attributable to ‘MLF+’ vs. ‘MLF−’ conditions.

### Planned Electrophysiological Data Analysis

The predictions specified in Table 2 refer to the complete adjective-noun constructions, and thus apply to responses measured on the final word within that construction. According to the MLF, the position of the adjective in relation to the noun should be congruent with the rules of the ML. In contrast, the MP model states that the position of the noun is contingent on the language of the adjective, irrespective of the ML. Thus, the model predictions may manifest differently in ERP effects elicited by nouns as compared to those elicited by adjectives. Separate EEG analyses were conducted for sentences in which the adjective-noun construction ended in an adjective and sentences in which the adjective-noun construction ended in a noun.

#### ERPs Time-Locked to Nouns (After an Adjective Has Already Been Presented)

The conditions included in this analysis are listed in Table 3. These sentences allow for a direct comparison since, by the time the noun is presented, the adjective has already been presented. As such, predictions can be made both in terms of MLF and MP as to the correct position of the noun, and violations would likely elicit a modulation of the Left Anterior Negativity (LAN, an ERP index considered to reflect early grammatical processing, with more negative amplitudes reflecting greater processing effort; Friederici et al., 1993). Thus, a 2 (MLF+ vs. MLF−) by 2 (MP+ vs. MP−) repeated measures ANOVA was conducted on five electrodes typically associated with the LAN (AF3, AF3, F7, F5, F3; Friederici et al., 1993). An additional exploratory ANOVA was conducted on six electrodes typically associated with the P600 (P1, PZ, P2, PO3, POZ, PO4).

**TABLE 3:** Experimental design and stimulus examples of sentences with a noun occurring after the adjective.

**Sentence**	**MLF**	**MP**
The girl bought one small **aderyn** *with her feet.*	**+**	**+**
The girl bought one bach **bird** during a shopping spree.	**+**	**–**
Prynodd y ferch un small **aderyn** ar ôl ysgol.	**–**	**+**
Prynodd y ferch un bach **bird** yn ystod gwyliau’r haf.	**–**	**–**
		

*The critical noun is highlighted in bold. An example of a semantically incongruous completion is highlighted in italics, though note that these incongruent completions were dispersed equally between conditions. Italics and bold font are for emphasis and were not used in the experiment.*

#### ERPs Time-Locked to Adjectives (After a Noun Has Already Been Presented)

The conditions included in this analysis are listed in Table 4. Given the specific predictions of both models, we assumed that noun presentation would not have allowed participants to generate any predictions regarding the position of the adjective. Upon reading the adjective, however, we propose that participants will have had to evaluate the appropriateness of its position by referring back to the noun. As such, violations would likely manifest in the range of the P600, a component typically involved in grammatical processing and re-evaluation, with more positive amplitudes reflecting greater processing and extent of re-evaluation (Osterhout and Holcomb, 1992; Steinhauer et al., 2009, 2010). Thus, a 2 (MLF+ vs. MLF−) by 2 (MP+ vs. MP−) repeated measures ANOVA was conducted on six electrodes typically associated with the P600 (P1, PZ, P2, PO3, POZ, PO4). An additional exploratory ANOVA was conducted on five electrodes typically associated with the LAN (AF3, AF3, F7, F5, F3; Friederici et al., 1993).

**TABLE 4:** Experimental design and stimulus examples of sentences with an adjective occurring after the noun.

**Sentence**	**MLF**	**MP**
The girl bought one bird **bach** from the pet store.	**–**	**+**
The girl bought one aderyn **small** without telling her parents	**–**	**–**
Prynodd y ferch un bird **bach** gyda ei phres poced.	**+**	**+**
Prynodd y ferch un aderyn **small** fel anhreg i’w chwaer.	**+**	**–**
		

*The target word is highlighted in bold. Italics and bold font are for emphasis and were not used in the experiment.*

### Extended Electrophysiological Data Analysis

In our extended analysis, invited by the reviewers of this paper, we compared ERPs elicited by the adjective within the noun phrase, regardless of whether it occurred before or after the noun (Table 5). This allowed us to include ML (Welsh vs. English) as an additional factor, and thus permits a more direct comparison to the production literature that has shown asynchronies in switching behaviours in different communities (Blokzijl et al., 2017). For this analysis, we again focused on the LAN and the P600, using the same time-window and electrodes as outlined above.

**TABLE 5:** Experimental design and stimulus examples.

**Sentence**	**MLF**	**MP**
The girl bought one bird **bach** from the pet store.	**–**	**+**
The girl bought one **small** aderyn *with her feet.*	**+**	**+**
The girl bought one aderyn **small** without telling her parents.	**–**	**–**
The girl bought one **bach** bird during a shopping spree.	**+**	**–**
Prynodd y ferch un bird **bach** gyda ei phres poced.	**+**	**+**
Prynodd y ferch un **small** aderyn ar ôl ysgol.	**–**	**+**
Prynodd y ferch un aderyn **small** fel anhreg i’w chwaer.	**+**	**–**
Prynodd y ferch un **bach** bird yn ystod gwyliau’r haf.	**–**	**–**

*In all cases, the model predictions are based on the placement of the adjective. The critical adjective is highlighted in bold. An example of a semantically aberrant sentence ending is highlighted in italics, though note that these incongruent completions were dispersed equally between conditions. Italics and bold font are for the reader’s information and were not used in the experiment.*

## Results

### Behavioural Analyses

#### Subjective Responses

**FIGURE 1:**
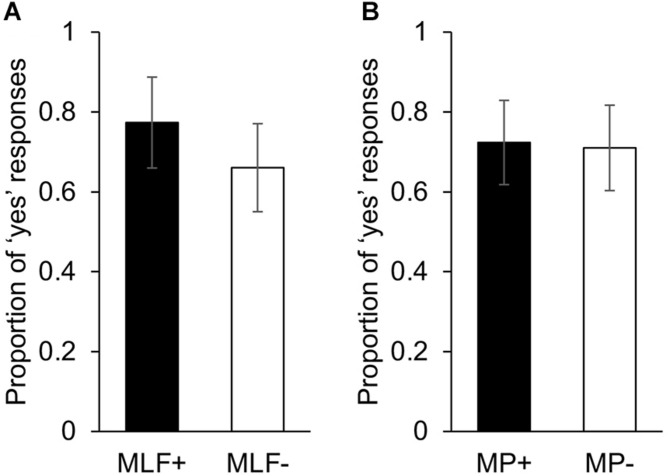
**(A)** Yes response ratios in the Semantic Congruency task for sentences that adhered to (MLF+), and violated (MLF–), the predictions of MLF. **(B)** Yes response ratios in the Semantic Congruency task for sentences that adhered to (MP+), and violated (MP–), the predictions of MP. Error bars represent the standard error of the mean. All the trials included in this analysis could have received a yes answer, since, from a ‘purely’ semantic viewpoint (that is, overlooking syntax), all sentences made sense.

The results of the binomial logistic regression can be seen in Table 6. A significant effect of MLF was found: Participants were more likely to state that a sentence made sense when MLF assumptions were met than when they were violated (Figure 1). In contrast, there was no significant effect of MP, nor was there a significant MLF^∗^MP interaction (MLF+MP+: *M* = 0.77, *SE* = 0.10; MLF+MP−: *M* = 0.78, *SE* = 0.10; MLF-MP+: *M* = 0.68, *SE* = 0.11; MLF−MP−: *M* = 0.64, *SE* = 0.11).

**TABLE 6:** Fixed effect estimates derived from the binomial logistic regression on subjective responses data.

	**Estimate**	**Standard Error**	***z* value**	***p*-value**
Intercept	1.21185	0.07162	16.92	<0.001
MLF	–0.47352	0.09627	–4.92	<0.001
MP	0.03275	0.10181	0.32	0.748
MLF*MP	–0.17655	0.13581	–1.30	0.194

#### Reaction Times

The results of the linear mixed effects analyses can be seen in Table 7. No significant differences were observed between sentences that adhered to MLF (*M* = 877, *SE* = 93) and sentences that violated MLF (*M* = 847, *SE* = 94). No significant differences were observed between sentences that adhered to MP (*M* = 867, *SE* = 92) and sentences that violated MP (*M* = 858, *SE* = 95). Finally, there was no significant MLF^∗^MP interaction (MLF+MP+: *M* = 887, *SE* = 91; MLF+MP−: *M* = 866, *SE* = 94; MLF−MP+: *M* = 846, *SE* = 92; MLF−MP−: *M* = 849, *SE* = 97).

**TABLE 7:** Fixed effect estimates derived from the linear mixed effects analysis on reaction time data.

	**Estimate**	**Standard Error**	***t*-value**
Intercept	895.14	45.78	19.55
MLF	–42.06	30.27	–1.39
MP	–18.61	28.55	–0.65
MLF*MP	22.74	39.06	0.58

*t > 1.96; *p* < 0.05.*

### Electrophysiological Results

**FIGURE 2:**
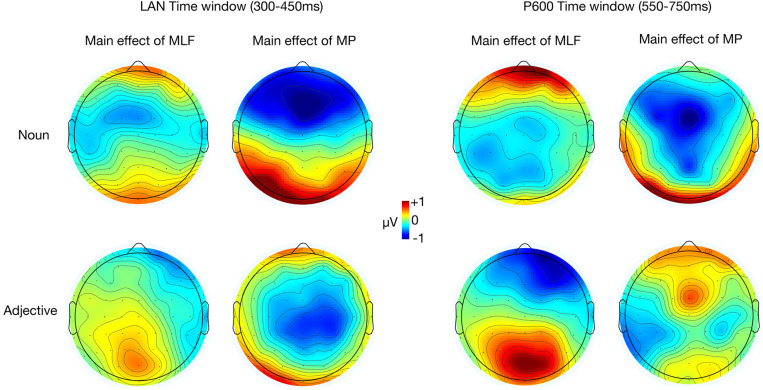
Topographic maps of ERP difference waves elicited by the adjective and the noun in the LAN and P600 analysis windows. Main effect of MLF depicts differences between sentences that violated MLF and sentences that adhered to MLF. Main effect of MP depicts differences between sentences that violated MP and sentences that adhered to MP.

Our electrophysiological analyses focused exclusively on two ERP components (LAN and P600) to ensure consistency with previous studies investigating the grammaticality of code-switches. Other studies have reported that morphosyntactic violations modulate N400 mean amplitude, however, scalp topographies from the current study support our *a priori* decision to focus on the LAN and P600 (Figure 2).

### Planned Analysis: ERPs Elicited by the Noun (Post Adjective Presentation)

**FIGURE 3:**
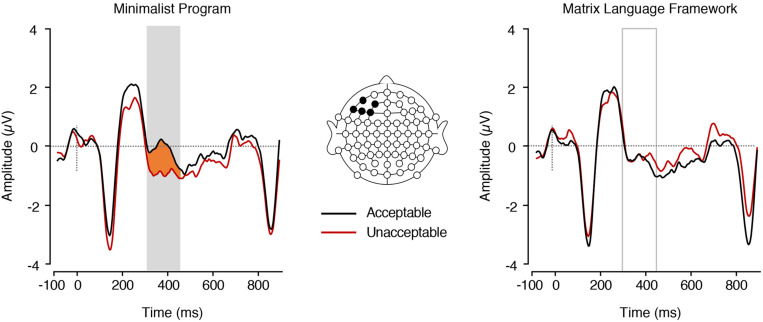
ERPs elicited by nouns preceded by adjectives. **Left**, in the MP+ and MP– conditions (main effect of MP). The plain grey box indicates the time window of the LAN analysis in which mean ERP amplitudes significantly differed between conditions (300–450 ms post-stimulus). **Right**, in the MLF+ and MLF- conditions (main effect of MLF).

A main effect of MP was found in the LAN range, *F*(1,17) = 9.94, *p* = 0.006,$\eta_{\text{p}}^{2}$ = 0.369, with nouns embedded in MP− sentences eliciting more negative mean ERP amplitudes (*M* = −1.58, *SE* = 0.34) than nouns embedded in MP+ sentences (*M* = −0.80, *SE* = 0.33; Figure 3). There was no significant difference between MLF+ sentences (*M* = −1.16, *SE* = 0.31) and MLF− sentences [*M* = −1.22, *SE* = 0.38; *F*(1,17) = 0.03, *p* = 0.857, $\eta_{\text{p}}^{2}$ = 0.002] nor a significant MLF^∗^MP interaction [*F*(1,17) = 1.48, *p* = 0.241, $\eta_{\text{p}}^{2}$ = 0.080; MLF+MP−: *M* = −0.64, *SE* = 0.33; MLF+MP−: *M* = −1.69, *SE* = 0.35; MLF−MP+: *M* = −0.97, *SE* = 0.42; MLF−MP−: *M* = −1.47, *SE* = 0.42].

**FIGURE 4:**
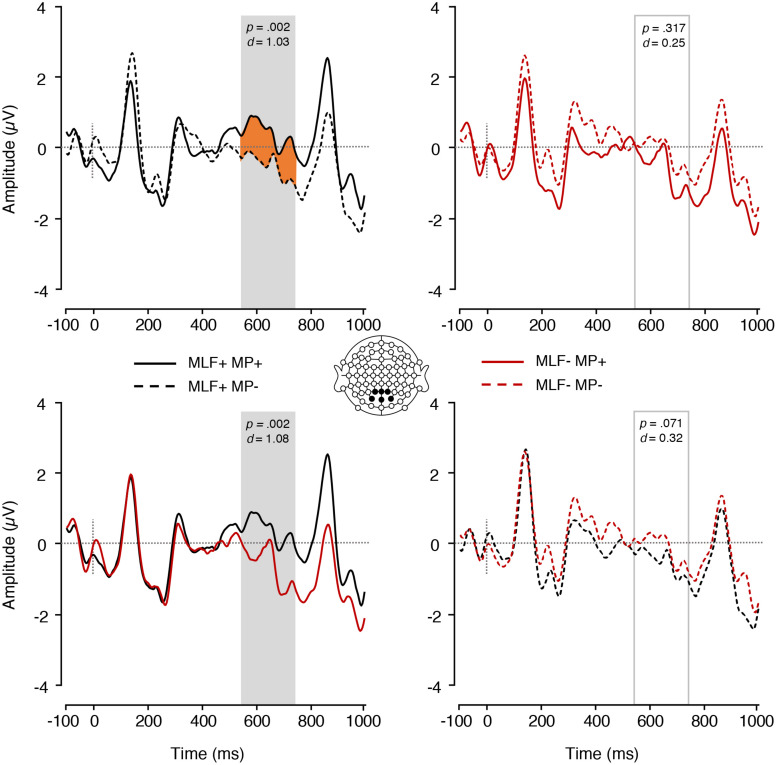
ERPs elicited by nouns preceded by adjectives depicting the interaction between MLF and MP. The plain grey box indicates the time window of the analysis in which mean ERP amplitudes significantly differed between conditions (550–750 ms post-stimulus). MLF, Matrix Language Framework; MP, Minimal Programme; +, stimuli compliant with prediction; –, stimulus violating prediction.

In the P600 range, no significant differences emerged between sentences that adhered to (*M = −*0.31, *SE* = 0.23) and sentences that violated (*M* = −0.65, *SE* = 0.25) the rules of MLF [*F*(1,17) = 2.99, *p* = 0.102, $\eta_{\text{p}}^{2}$ = 0.149]. Similarly, no significant differences emerged between sentences that adhered to (*M* = −0.25, *SE* = 0.19) and violated (*M* = −0.72, *SE* = 0.31) the rules of MP [*F*(1,17) = 3.23, *p* = 0.090, $\eta_{\text{p}}^{2}$ = 0.160]. However, a significant MLF^∗^MP interaction did emerge [*F*(1,17) = 18.08, *p* = 0.001, $\eta_{\text{p}}^{2}$ = 0.515; Figure 4]. Paired samples *t*-tests were conducted to tease apart the interaction, and an adjusted significance threshold of 0.013 was used to reduce the possibility of a Type I error. When MP predictions were adhered to, sentences that adhered to MLF elicited more positive mean amplitudes (*M* = 0.31, *SE* = 0.21) than sentences that violated MLF [*M* = −0.80, *SE* = 0.27; *t*(17) = 3.73, *p* = 0.002, *d* = 1.08]. Similarly, when MLF predictions were adhered to, sentences that adhered to MP predictions elicited more positive mean amplitudes (*M* = 0.31, *SE* = 0.21) than sentences that violated MP predictions [*M* = −0.94, *SE* = 0.35; *t*(17) = 3.70, *p* = 0.002, *d* = 1.03]. No other significant effects were found.

### Planned Analysis: ERPs Elicited by the Adjective (Post Noun Presentation)

In the LAN time-window, there was no main effect of MLF [*F*(1,17) = 0.02, *p* = 0.905, $\eta_{\text{p}}^{2}$ = 0.001], no main effect of MP [*F*(1,17) = 0.05, *p* = 0.819, $\eta_{\text{p}}^{2}$ = 0.003], and no significant MLF^∗^MP interaction [*F*(1,17) = 0.001, *p* = 0.972, $\eta_{\text{p}}^{2}$ < 0.001].

**FIGURE 5:**
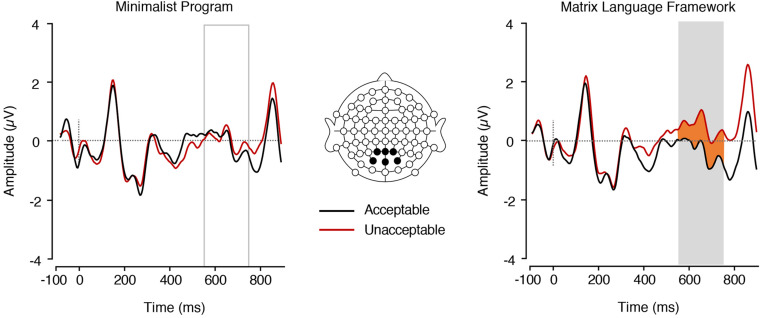
ERPs elicited by adjectives preceded by nouns. **Left**, in the MP+ and MP– conditions (main effect of MP). **Right**, in the MLF+ and MLF– conditions (main effect of MLF). The plain grey box indicates the time window of the analysis in which mean ERP amplitudes significantly differed between conditions (550–750 ms post-stimulus).

We found a significant main effect of MLF in the P600 range, *F*(1,17) = 11.04, *p* = 0.004, $\eta_{\text{p}}^{2}$ = 0.394, with adjectives embedded in MLF- sentences eliciting more positive mean amplitudes (*M* = 0.45 *SE* = 0.28) than adjectives embedded in MLF+ sentences (*M* = −0.36, *SE* = 0.21; Figure 5).

**FIGURE 6:**
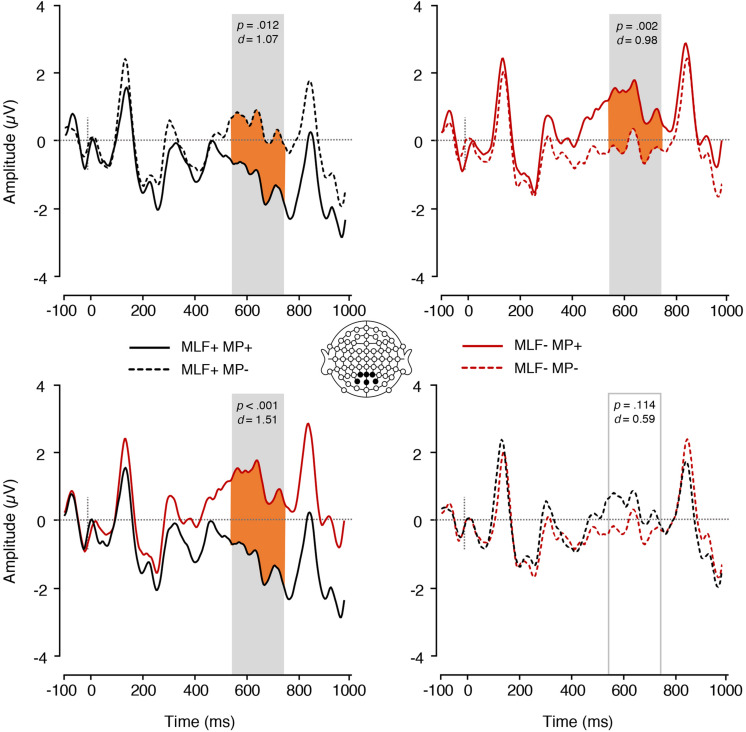
ERPs elicited by adjectives preceded by nouns, depicting the interaction between MLF and MP. The plain grey box indicates the time window of the analysis in which mean ERP amplitudes significantly differed between conditions (550–750 ms post-stimulus). MLF, Matrix Language Framework; MP, Minimal Programme; +, stimuli compliant with prediction; –, stimulus violating prediction.

We also found a significant interaction between MP and MLF in the P600 range [*F*(1,17) = 14.31, *p* = 0.001, $\eta_{\text{p}}^{2}$ = 0.46; Figure 6]. Paired samples *t*-tests revealed that, for MLF+ sentences, MP violations elicited more positive mean amplitudes (*M* = 0.43, *SE* = 0.37) than MP compliances [*M* = −1.16, *SE* = 0.33; *t*(17) = −2.80, *p* = 0.012, *d* = 1.07], whereas MLF- sentences showed the reverse pattern, with MP violations eliciting more negative mean amplitudes (*M* = −0.26, *SE* = 0.28) than MP compliances [*M* = 1.16, *SE* = 0.39; *t*(17) = 3.68, *p* = 0.002, *d* = 0.98]. In addition, for MP+ sentences, MLF violations elicited more positive mean amplitudes (*M* = 1.16, *SE* = 0.39) than MLF adherences [*M* = −1.16, *SE* = 0.33; *t*(17) = −4.50, *p* < 0.001, *d* = 1.51]. For MP− sentences however, no significant difference was observed between the MLF+ and MLF− conditions, *t*(17) = 1.67, *p* = 0.114, *d* = 0.59. No significant main effect of MP was found, *F*(1,17) = 0.09, *p* = 0.765, $\eta_{\text{p}}^{2}$ = 0.005.

### Extended Analysis: ERPs Elicited by Adjectives Regardless of Position

In the LAN time window, a 2 (Matrix Language: English vs. Welsh) × 2 (MLF+ vs. MLF−) × 2 (MP+ vs. MP−) repeated measures ANOVA revealed a significant main effect of MLF [*F*(1,17 = 5.45, *p* = 0.032, $\eta_{\text{p}}^{2}$ = 0.243], with sentences that violated the predictions of MLF eliciting more negative mean amplitudes (*M* = −1.02, *SE* = 0.38) than sentences that adhered to the predictions of MLF (*M* = −0.40, *SE* = 0.32). No other significant effects were found.

In the P600 window, a 2 (Matrix Language: English vs. Welsh) × 2 (MLF+ vs. MLF−) × 2 (MP+ vs. MP−) repeated measures ANOVA revealed a significant main effect of MLF [*F*(1,17 = 13.25, *p* = 0.002, $\eta_{\text{p}}^{2}$ = 0.438], with sentences that violated the predictions of MLF eliciting more positive mean amplitudes (*M* = 0.95, *SE* = 0.20) than sentences that adhered to the predictions of MLF (*M* = 0.17, *SE* = 0.20). There were no other significant main effects [Main effect of Matrix Language: *F*(1,17) = 0.02, *p* = 0.900, $\eta_{\text{p}}^{2}$ = 0.001; Main effect of MP: *F*(1,17) = 0.21, *p* = 0.653, $\eta_{\text{p}}^{2}$ = 0.012].

**FIGURE 7:**
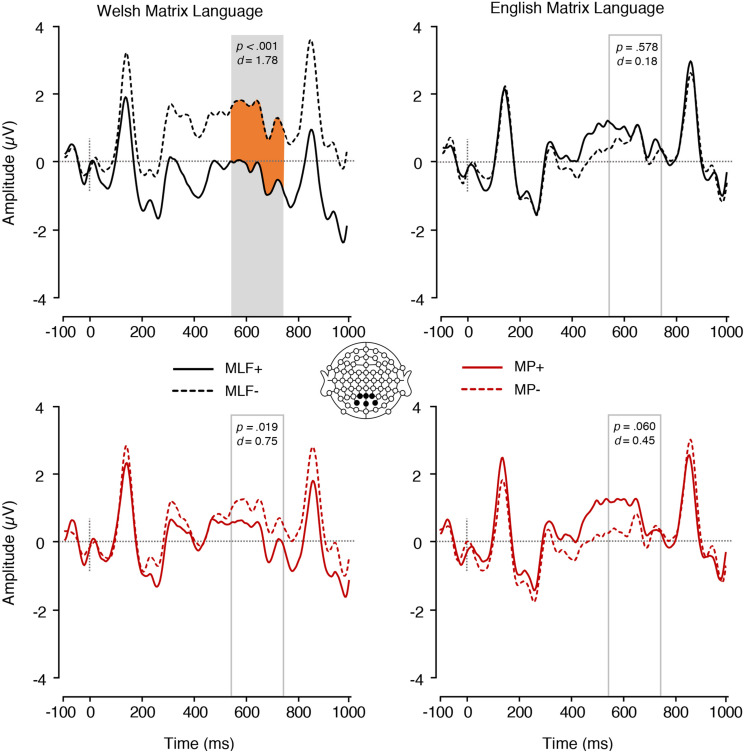
ERPs elicited by adjectives depicting the Matrix language^∗^MLF and the Matrix language^∗^MP interactions. The plain grey box indicates the time window of the analysis in which mean ERP amplitudes significantly differed between conditions (550–750 ms post-stimulus). MLF, Matrix Language Framework; MP, Minimalist Programme; +, stimuli compliant with prediction; –, stimulus violating prediction.

The Matrix language^∗^MLF interaction was, however, significant [*F*(1,17) = 9.44, *p* = 0.007, $\eta_{\text{p}}^{2}$ = 0.357; Figure 7]. Paired samples *t*-tests were conducted to tease apart the interaction, and an adjusted significance threshold of 0.013 was used to reduce the possibility of a Type I error. When the ML was Welsh, sentences that violated the predictions of MLF elicited more positive mean amplitudes (*M* = 1.45, *SE* = 0.27) than sentences that adhered to the predictions of MLF [*M* = −0.36, *SE* = 0.21; *t*(17) = −5.07, *p* < 0.001, *d* = 1.78]. However, when the ML was English, no significant difference emerged between sentences that violated (*M* = 0.45, *SE* = 0.28) and sentences that adhered to the predictions of MLF [*M* = 0.70, *SE* = 0.37; *t*(17) = 0.57, *p* = 0.578, *d* = 0.18]. Sentences that adhered to the predictions of MLF elicited more positive mean amplitudes when the ML was English (*M* = 0.70, *SE* = 0.37) than when the ML was Welsh [*M* = −0.36, *SE* = 0.21; *t*(17) = −2.44, *p* = 0.026, *d* = 0.84]. However, sentences that violated the predictions of MLF elicited more positive mean amplitudes when the ML was *Welsh* (*M* = 1.45, *SE* = 0.27) than when the ML was English [*M* = 0.45, *SE* = 0.28; *t*(17) = 2.58, *p* = 0.020, *d* = 0.85].

We also found a significant Matrix language ^∗^ MP interaction [*F*(1,17) = 8.36, *p* = 0.010, $\eta_{\text{p}}^{2}$ = 0.330; Figure 7]. Paired samples *t*-tests were conducted to tease apart the interaction, and an adjusted significance threshold of 0.013 was used to reduce the possibility of a Type I error. When the ML was Welsh, a trend was observed, with sentences that violated the rules of MP eliciting more positive mean amplitudes (*M* = 0.87, *SE* = 0.25) than sentences that adhered to the rules of MP [*M* = 0.21, *SE* = 0.14; *t*(17) = −2.60, *p* = 0.019, *d* = 0.75]. However, when the ML was English, no significant difference was found between sentences that violated (*M* = 0.31, *SE* = 0.23) and sentences that adhered to the rules of MP [*M* = 0.83, *SE* = 0.32; *t*(17) = 2.02, *p* = 0.060, *d* = 0.45]. When MP rules were followed, more positive mean amplitudes were elicited when the ML was English (*M* = 0.83, *SE* = 0.32) than when the ML was Welsh (*M* = 0.21, *SE* = 0.14), though this difference was not significant [*t*(17) = −1.89, *p* = 0.076, *d* = 0.60]. When MP rules were violated, more positive mean amplitudes were elicited when the ML was Welsh (*M* = 0.87, *SE* = 0.25) than when the ML was English (*M* = 0.31, *SE* = 0.23), though this difference was not significant [*t*(17) = 1.86, *p* = 0.080, *d* = 0.55].

**FIGURE 8:**
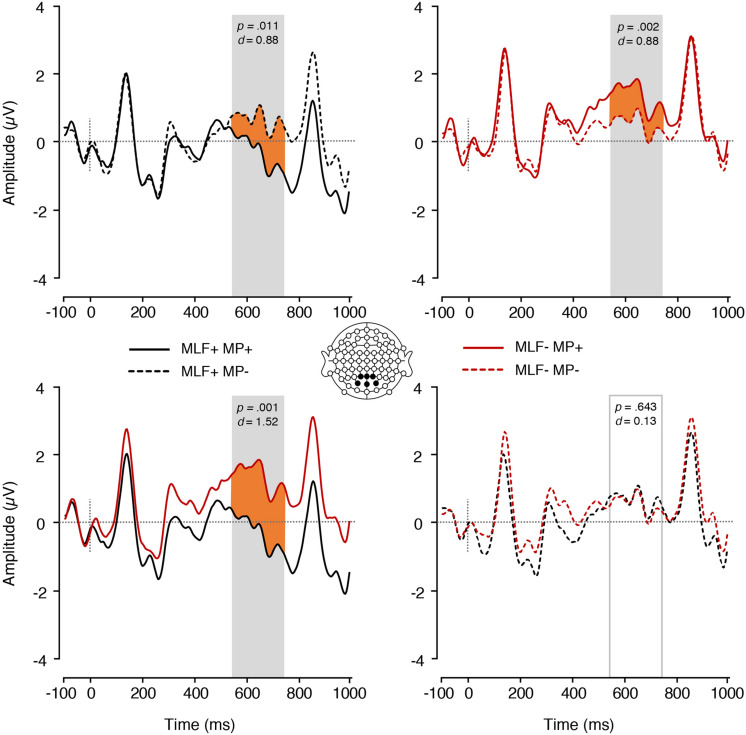
ERPs elicited by all adjectives, depicting the interaction between MLF and MP. The plain grey box indicates the time window of the analysis in which mean ERP amplitudes significantly differed between conditions (550–750 ms post-stimulus). MLF, Matrix Language Framework; MP, Minimalist Programme; +, stimuli compliant with prediction; –, stimulus violating prediction.

Finally, a significant MLF^∗^MP interaction was found [*F*(1,17) = 13.50, *p* = 0.002, $\eta_{\text{p}}^{2}$ = 0.443; Figure 8]. Paired samples *t*-tests were conducted to tease apart the interaction, and an adjusted significance threshold of 0.013 was used to reduce the possibility of a Type I error. For MLF+ sentences, MP violations elicited more positive mean amplitudes (*M* = 0.66, *SE* = 0.27) than MP compliances [*M* = −0.32, *SE* = 0.26; *t*(17) = −2.84, *p* = 0.011, *d* = 0.88]. However, MLF- sentences showed the reverse pattern, with MP violations eliciting more negative mean amplitudes (*M* = 0.53, *SE* = 0.18) than MP compliances [*M* = 1.37, *SE* = 0.26; *t*(17) = 3.70 *p* = 0.002, *d* = 0.88]. In addition, for MP+ sentences, MLF violations elicited more positive mean amplitudes (*M* = 1.37, *SE* = 0.26) than MLF adherences [*M* = −0.32, *SE* = 0.26; *t*(17) = −4.47, *p* < 0.001, *d* = 1.52]. For MP− sentences, however, no significant difference was observed between the MLF+ and MLF− conditions, *t*(17) = 0.47, *p* = 0.643, *d* = 0.13. No other significant effects were found.

## Discussion

In this study, we investigated the predictions of two theoretical accounts of code-switching in a real time word-by-word reading context. We asked Welsh–English bilingual participants to read Welsh and English sentences that contained a code-switch that either adhered to the predictions of both the MLF (Myers-Scotton, 1993) and the MP (Cantone and MacSwan, 2009), violated the predictions of both accounts, or violated the predictions of one account but complied with the predictions of the other. On-line processing of the code-switches was assessed using ERPs elicited by nouns and adjectives, with violations eliciting greater ERP mean amplitudes in the time windows of the LAN and the P600. These components reflect two separate analyses and provide complementary findings. Both components are discussed independently below before we provide an integrated discussion of our findings. Participant responses in a semantic AJT were also used as an indirect measure of the model predictions at a surface level, i.e., more explicit and metacognitive in nature.

The LAN findings from our analyses on the noun lend support to MP, since sentences that violated MP rules elicited greater mean LAN amplitudes as compared to sentences that complied with MP, and thus they required greater cognitive processing (Friederici et al., 1993; Hahne and Friederici, 1999; Friederici, 2002; Hagoort, 2003; Steinhauer et al., 2009, 2010). In contrast, MLF violation or compliance did not elicit measurable ERP modulations in the LAN time-window. However, in our additional analyses which focused on the adjective in all experimental sentences, irrespective of placement (pre- or post-nominally), we found support for the MLF over the MP. Here, sentences that violated the predictions of MLF required greater processing effort than sentences that adhered to the predictions of MLF, whilst no difference was observed between sentences that violated and adhered to the predictions of MP. Given that the LAN is assumed to reflect early parsing mechanisms and morphosyntactic analysis (Hahne and Friederici, 1999; Steinhauer et al., 2009, 2010), it is possible that both MP and MLF predictions are relevant for local-level grammatical processing. However, we note that the predictions of both models are based on adjective position, and that the data supporting MP were elicited by nouns immediately following an adjective (see Table 3), whilst the data supporting MLF were elicited by adjectives in pre- and post-nominal position (see Tables 4, 5).

Our P600 findings show a markedly different pattern of results, which favour the predictions of the MLF and require a dedicated interpretation. Here, sentences that adhered to the predictions of MLF (MLF+ sentences) elicited attenuated P600 mean amplitudes as compared to sentences that violated them. Thus, violations of MLF elicited greater processing and re-evaluation than MLF orthodox sentences, providing support for the findings of Parafita Couto et al. (2017). However, MP predictions did not elicit modulations in the P600 range, suggesting that, on a global, sentence level, MLF predictions prevail. This finding was consistent across all our analyses, thus providing strong support for MLF over MP. Given the nature of the models themselves, such a finding does not seem unreasonable, since MLF predictions are based on the ML of clauses, which requires an analysis extending well beyond single word processing. This interpretation is further consistent with the observation that MLF predictions, not MP ones, aligned with the proportions of ‘yes’ responses provided by participants in the semantic acceptability judgement task. In other words, if the sentence as a whole seemed acceptable from a syntactic point of view based on the MLF (MLF+ sentences in Table 2), not only did critical words require less re-evaluation than for those sentences that violated MLF (MLF− sentences in Table 2), but also the sentence was more likely to be judged as semantically acceptable.

We also found significant MLF^∗^MP interactions in the P600 time-window, with differing patterns depending on whether the analysis was time-locked to the noun or the adjective. When measuring ERPs on the final noun within the adjective-noun construction, *post hoc* comparisons revealed an unexpected pattern: When dealing with sentences that adhered to MLF (MLF+), those adhering to MP predictions required *more* processing effort than those that violated MP predictions. Similarly, when dealing with sentences that adhered to MP (MP+), those adhering to MLF predictions required *more* processing effort than those that violated MLF predictions. These findings are counterintuitive, and do not align with the predictions of either model. One *post hoc* explanation is that differences in the placement of the code-switched word triggered this effect. In all cases, sentences that adhered to the predictions of both models included a noun insertion following the adjective (e.g., The girl bought one small **aderyn**), whilst sentences that adhered to the predictions of one model but not the other contained a ‘double-switch,’ where an adjective insertion occurred before the noun where the measurement took place (e.g., The girl bought one **bach** bird; Prynodd y ferch un **small** aderyn). It is possible therefore that the greater processing difficulty observed in the MLF+MP+ condition may in fact reflect a switching cost (see Van Hell et al., 2015, 2018), rather than an implicit assessment of the predictions of MP and MLF.

When measuring ERPs on the adjective, *post hoc* comparisons revealed two intuitive and two intriguing findings: When dealing with sentences that adhered to MLF (MLF+), those adhering to MP predictions (MP+; 5a and 5b) required less processing effort than those that violated MP (MP−; 5c and 5d).

(5) (a) Prynodd y ferch un bird bach gyda ei phres poced(MLF+ MP+)
(b) The girl bought one small aderyn with her feet(MLF+ MP+)
(c)  Prynodd y ferch un aderyn small fel anrheg i’w chwaer(MLF+MP−)
(d)  The girl bought one bach bird during a shopping spree(MLF+ MP−)

When focusing on sentences that adhered to MP (MP+), sentences that also adhered to MLF (MLF+; 6a and 6b) required less processing effort than those that violated MLF (MLF−; 6c and 6d).

(6) (a) Prynodd y ferch un bird bach gyda ei phres poced(MLF+ MP+)
  (b) The girl bought one small aderyn with her feet(MLF+ MP+)
  (c) The girl bought one bird bach from the pet store(MLF− MP+)
  (d) Prynodd y ferch un small aderyn ar ôl ysgol(MLF− MP+)

That is, sentences that adhered to the predictions of both models required less processing effort. It is worth noting, however, that all sentences that adhered to both models (5a, 5b; 6a, 6b) included a noun insertion (which are frequent in naturalistic production; cf. Parafita Couto et al., 2015) and all sentences that violated one model but adhered to the other (5c, 5d; 6c, 6d) contained an adjective insertion (which are infrequent in production). Finally, when focusing on sentences violating MLF (MLF−), MP violations (MP−; 7a, 7b) are easier to process than MP compliant stimuli (MP+; 7c, 7d). That is, sentences that violated both models (MLF−MP−) required less processing effort than sentences that violated the rules of MLF but adhered to the rules of MP (MLF−MP+).

(7) (a) The girl bought one aderyn small without telling herparents (MLF−MP−)
  (b) Prynodd y ferch un bach bird yn ystod gwyliau’rhaf (MLF−MP−).
  (c) The girl bought one bird bach from the pet store(MLF−MP+)
  (d) Prynodd y ferch un small aderyn ar ôlysgol (MLF− MP+).

While this finding does not straightforwardly match the predictions of either model (both models would predict the pattern observed for 5a, 5b, 6a, 6b, 7a, 7b, but differ in their prediction for 5c, 5d, 6c, 6d, 7c, 7d), such a finding is consistent with previous AJT and production studies (e.g., Stadthagen-González et al., 2017; Voss, 2018; Parafita Couto and Gullberg, 2019), and may reflect a general preference for noun insertions over adjective insertions. Indeed, all sentences violating both MLF and MP (e.g., 7a) featured a noun insertion, whilst sentences violating MLF but not MP (e.g., 7b) all featured an adjective insertion (Tables 4, 7). Finally, when sentences violated MP, we found no significant differences between sentences that violated MLF (MP−MLF−) and sentences that adhered to its predictions (MP−MLF+). We speculate that this may provide additional support for MLF over MP, as sentences that adhered to MLF but not MP are processed with the same ease as sentences that violated the predictions of both models and included noun insertions. We tentatively suggest that this null effect highlights a similar preference for MLF+MP− sentences, thus providing support for MLF. This suggestion is strengthened as the previous comparison revealed that MLF−MP+ sentences required *more* processing effort than MLF−MP− sentences. As such, it appears as though the impact of MP is minimal at a global processing level.

Our additional analyses also revealed that MLF and MP predictions manifest differently depending on the ML of the sentence (Welsh or English): When the ML was Welsh, sentences that violated the predictions of MLF required greater processing effort than sentences that adhered to the predictions of MLF. Similarly, when the ML was Welsh, sentences that violated the predictions of MP required greater processing effort than sentences that adhered to MP predictions. However, when the ML was English, ERP responses were not significantly modulated by neither MLF nor MP predictions. This asymmetry cannot be attributed to noun insertion preference, and so an alternative interpretation is required. A consistent finding in the corpus literature is that code-switches are more prevalent in one language over the other (e.g., in Parafita Couto et al., 2015, Welsh was the ML for all sentences that contained a code-switch, with English being the EL- sentences with English as the ML and Welsh as the EL were unattested), and so this asynchrony may reflect community characteristics that are specific to this population. In fact, Valdés Kroff (2016) posited, based on Spanish-English data, that code- switching is a learned behaviour, which may vary from community to community, an assumption that is consistent with psycholinguistic models that suggest that processing patterns are impacted by statistical regularities observed in production (e.g., MacDonald, 2013; Pickering and Garrod, 2013; Dell and Chang, 2014). He suggested that the profile of the bilinguals in terms of usage and exposure to code-switching should result in observable group differences, both in the production and comprehension of code-switching. In the case of the Welsh–English community, code-switched constructions may be more common when the ML is Welsh, leading participants to generate stronger expectations about the placement of the code-switch. When the ML is English, however, such expectations may not apply, due to the infrequent occurrence of Welsh insertions into English sentences. This finding could also explain some of the conflicting patterns observed in previous electrophysiological studies, which may not have considered the ML of the sentence as a confounding factor within their analyses (Parafita Couto et al., 2017; Pablos et al., 2018). We therefore suggest that any future studies assessing the predictions of MLF and MP include the ML of the sentence as an experimental factor (Stadthagen-González et al., 2017; cf. Parafita Couto and Stadthagen-Gonzalez, 2019).

This effect could also be a result of syntactic co-activation. A substantial body of evidence suggests that bilinguals automatically activate the syntactic rules of both their languages, even when they operate in a single language context (e.g., Hartsuiker et al., 2004; Scheutz and Eberhard, 2004; Desmet and Declercq, 2006; Hartsuiker and Pickering, 2008; Lemhöfer et al., 2008; Weber and Indefrey, 2009; Paolieri et al., 2010; Ganushchak et al., 2011; Hatzidaki et al., 2011; Sanoudaki and Thierry, 2014; Vaughan-Evans et al., 2014; Sanoudaki and Thierry, 2015; Bernolet and Hartsuiker, 2018). Whilst some studies have suggested that similarity in syntactic structure across languages can determine the degree of syntactic co-activation (Loebell and Bock, 2003; Bernolet et al., 2007; Kantola and van Gompel, 2011; Kidd et al., 2015), neuroscientific investigations of cross-language syntactic activation have shown that idiosyncratic rules (e.g., Vaughan-Evans et al., 2014) and syntactic rules conflicting across language such as word-order (e.g., Sanoudaki and Thierry, 2014, 2015) are also the object of automatic co-activation. It is possible that, when reading sentences with an English ML, our participants automatically activated and applied the grammatical rules of Welsh, that stipulate that an adjective should occur in post-nominal position (though see Borsley et al., 2007 for counterexamples). For example, when the ML was English, adjectives in pre-nominal positions were classed as grammatically correct according to the predictions of MLF, however, activation of the Welsh grammatical rules would deem such utterances as grammatically incorrect. Conversely, adjectives in post-nominal positions were classed as grammatically incorrect according to the predictions of MLF, yet activation of the Welsh grammatical rules would classify such utterances as grammatically correct. As such, any impact of MLF may have been ‘cancelled out’ in these sentences. The same rationale could be applied when considering MP predictions, thus providing a possible explanation for the null effect. A similar argument could be made when considering the Welsh ML sentences, however, studies have demonstrated that co-activation of L2 syntax during L1 processing is comparably weaker than the activation of L1 syntax during L2 processing (e.g., Hatzidaki et al., 2011). As such, the conflicting grammatical rules of English may not have been activated to such a degree that they counteracted the predictions of MP and MLF. We acknowledge that such an interpretation is *post hoc*, and reiterate that the purpose of this study was to assess the predictions of two competing linguistic models (MLF vs. MP) rather than to investigate syntactic co-activation. Future studies should, however, take this factor into consideration when assessing code-switching patterns.

Our findings expand upon two previous ERP studies that attempted to evaluate the competing predictions of MP and MLF (Parafita Couto et al., 2017; Pablos et al., 2018). Methodological differences as well as decisions relating to statistical analyses could provide an explanation for any discrepancies. Specifically, the support provided for MP in this study is derived from analyses time-locked to the onset of the noun, an analysis that was not conducted in the previous studies. Support for MLF, however, stems from analyses time-locked to the onset of the adjective and is in keeping with the analyses performed in the previous two papers. This raises an important practical question about the best way to measure the acceptability of code-switching patterns in neuroscientific studies, particularly when the two languages have conflicting word orders: Should all analyses be conducted on the code-switched word, should all analyses be conducted on the adjective within the noun phrase, or should all analyses be conducted on the final word within the adjective-noun construction? We initially argued for the latter, given that the predictions outlined in Table 2 refer to the position of the adjective in relation to the noun, and as such, participants would need to process the noun phrase in its entirety to determine the appropriateness of the code-switch. However, additional analyses focussing on the adjective across all experimental sentences allowed for a direct comparison of the sentence MLs (Welsh vs. English), which was not possible in the analysis testing our initial hypotheses. We do not provide a definitive answer here, but we encourage researchers investigating this empirical question in the future to consider this issue carefully, and to clearly outline and justify the comparisons made.

Our experimental design and the selected comparisons allowed for the analysis of an additional ERP component, the P600. Whilst these findings are not directly comparable to the findings of Parafita Couto et al. (2017) and Pablos et al. (2018), they provide insight into the complexities of the rules that govern code-switches. Our findings in relation to the P600 provide partial support for the findings of Parafita Couto et al. (2017), as sentences violating the predictions of MLF required greater processing effort than sentences adhering to its predictions. Our P600 findings also replicate the findings of previous papers (e.g., Stadthagen-González et al., 2017) as participants demonstrated a general preference for sentences that adhered to the predictions of both models (MLF+MP+) over sentences that adhered to the predictions of one model but not the other (e.g., MLF+MP−; MLF-MP+). Finally, our findings highlight a possible preference for noun insertions over adjective insertions, in line with previous findings (e.g., Stadthagen-González et al., 2017; Voss, 2018; Parafita Couto and Gullberg, 2019). However, we note that this interpretation does not account for the preference toward sentences that adhered to MLF over sentences that violated its rules, as both sentence types (MLF+ and MLF−) included both noun and adjective insertions.

As previously suggested (e.g., Stadthagen-González et al., 2017), our results do not lend support to the suggestion that it is only one of the theoretical proposals (either the ML or the language of the adjective) that regulates the relative order of adjectives and nouns in code-switched nominal constructions. Santorini and Mahootian (1995) and Mahootian and Santorini (1996) proposed all combinations of adjectives and nouns are possible. In line with Pablos et al. (2018), however, our data do not support this earlier proposal either. Rather, our ERP findings provide initial evidence to validate the predictions of both the MP and MLF theoretical accounts, with arguably stronger evidence in favour of MLF. Based on our findings in relation to LAN, MP and MLF predict local grammatical processing (word level integration in the syntactic frame sensitive to morphosyntatic processing). Note, however, that support for MP derives from ERPs elicited on the final noun within a noun-phrase, whilst support for MLF derives from ERPs elicited on the adjective within the noun-phrase. Whilst both models affected our P600 data, we argue that the impact of MP at this level represents a general preference for noun over adjective-insertions and thus argue that MLF predicts global syntactic integration and evaluation mechanisms (the impact of word integration on sentence-level processing). Critically, the behavioural data collected online are consistent with such an interpretation, since participant judgements, like P600 amplitudes, were only affected by MLF predictions. Our findings therefore suggest that the predictions of MLF primarily contribute to determining a felicitous code-switch, though analyses conducted on nouns also provide tentative support for the predictions of MP (see also Stadthagen-González et al., 2017).

At the same time, our results reflect the switching pattern that has previously been reported in naturalistic production in this bilingual community (Parafita Couto et al., 2015), i.e., a preference for noun (rather than adjective) insertions. This highlights the importance of studying code-switching from a language ecological perspective, as our results lend support to the claim that processing of code-switched structures should reflect context-specific patterns that reveal themselves both in production and in grammatical intuitions (e.g., Beatty-Martínez et al., 2018; Balam et al., 2020). Crucially, this preference for noun insertions has also been observed in other bilingual communities (Spanish–English, Papiamento–Dutch), both in production and AJT studies (Gullberg and Parafita Couto, 2016; Stadthagen-González et al., 2017; Voss, 2018; Balam and Parafita Couto, 2019). If this tendency is further confirmed in other bilingual communities, the fact that both theoretical proposals seem to be contributing to determining noun-adjective code-switching may just be a by-product of this general tendency in use. Instead, these findings could be taken as support for Backus’ (2014) suggestion that ¨the field of code-switching studies could be reinvigorated by the introduction of a usage-based approach¨ (p.19).

Overall, we have illustrated how the use of a hypothetico-deductive approach can unravel the complexities of intra-sentential code-switching, and we hope to have helped build a bridge between theoretically, and psycholinguistically driven studies on code-switching. The electrophysiological technique outlined in the present study can complement corpus and behavioural approaches with ¨an eye toward separating quasi-universal from language-specific code-switching configurations¨ (cf. Lipski, 2019, p. 23). The extension of bilingual language processing research to include other language combinations as well as other switch points holds the promise of refining our theoretical understanding of the rules governing intra-sentential code-switching.

## Data Availability Statement

The raw data supporting the conclusions of this article will be made available by the authors, without undue reservation.

## Ethics Statement

The studies involving human participants were reviewed and approved by the Bangor University School of Psychology ethics committee. The patients/participants provided their written informed consent to participate in this study.

## Author Contributions

All the authors conceived the study, contributed to the research design and manuscript revision, read and approved the submitted version. AV-E collected and analysed the data. AV-E, GT, and MP were responsible, in consultation with PW-D and MD, for the theoretical interpretation of the data. MP and AV-E wrote the manuscript.

## Conflict of Interest

The authors declare that the research was conducted in the absence of any commercial or financial relationships that could be construed as a potential conflict of interest.

## Footnotes

1 All of the predictions outlined above are based on two theoretical models (MLF and MP), as our goal was to empirically test the predictions of both models. The two models focus exclusively on grammaticality, and do not take into account extraneous factors such as frequency of usage in their predictions about the relative order of adjectives and nouns in switched nominal constructions. As such, the predictions outlined here do not consider the impact of such factors, however, we acknowledge and discuss the potential impact of these factors in the section “Discussion.”
